# Hand Motion Analysis during the Execution of the Action Research Arm Test Using Multiple Sensors

**DOI:** 10.3390/s22093276

**Published:** 2022-04-24

**Authors:** Jesus Fernando Padilla-Magaña, Esteban Peña-Pitarch, Isahi Sánchez-Suarez, Neus Ticó-Falguera

**Affiliations:** 1 Escola Politècnica Superior d’Enginyeria de Manresa (EPSEM), Polytechnic University of Catalonia, 08242 Manresa, Barcelona, Spain; esteban.pena@upc.edu; 2 Department of Manufacturing Technologies, Polytechnic University of Uruapan Michoacán, Uruapan 60210, Michoacán, Mexico; isahi_ss@hotmail.com; 3 Physical Medicine and Rehabilitation Service, Althaia Xarxa Assistencial de Manresa, 08243 Manresa, Barcelona, Spain; 29346ntf@gmail.com

**Keywords:** finger joints, flexion angle, fingertip force, action research arm test, hand

## Abstract

The Action Research Arm Test (ARAT) is a standardized outcome measure that can be improved by integrating sensors for hand motion analysis. The purpose of this study is to measure the flexion angle of the finger joints and fingertip forces during the performance of three subscales (Grasp, Grip, and Pinch) of the ARAT, using a data glove (CyberGlove II^®^) and five force-sensing resistors (FSRs) simultaneously. An experimental study was carried out with 25 healthy subjects (right-handed). The results showed that the mean flexion angles of the finger joints required to perform the 16 activities were Thumb (Carpometacarpal Joint (CMC) 28.56°, Metacarpophalangeal Joint (MCP) 26.84°, and Interphalangeal Joint (IP) 13.23°), Index (MCP 46.18°, Index Proximal Interphalangeal Joint (PIP) 38.89°), Middle (MCP 47.5°, PIP 42.62°), Ring (MCP 44.09°, PIP 39.22°), and Little (MCP 31.50°, PIP 22.10°). The averaged fingertip force exerted in the Grasp Subscale was 8.2 N, in Grip subscale 6.61 N and Pinch subscale 3.89 N. These results suggest that the integration of multiple sensors during the performance of the ARAT has clinical relevance, allowing therapists and other health professionals to perform a more sensitive, objective, and quantitative assessment of the hand function.

## 1. Introduction

The human hand is one of the most complex and fascinating structures in the human body consisting of 27 bones, including 8 carpal bones, 5 metacarpals, and 14 phalanges, making it difficult to study [1]. Human hand function allows object manipulation and physical interaction with the environment. Deficits in hand function severely affect their quality of life by preventing them from performing activities of daily living (ADLs). Stroke is the number one cause of severe adult disability in the U.S. and worldwide [2,3]. One of the main sequels after a stroke is the loss of mobility in the upper extremities, often affecting hand dexterity. Therefore, the rehabilitation process for hand recovery is vital to post-stroke patients. The rehabilitation process is traditionally carried out by a physical therapist specializing in treating disabilities related to motor and sensory impairments [4]; hand therapy helps to improve strength and increase the range of motion (ROM) extension/flexion. Therefore, to evaluate and improve the efficacy of rehabilitation programs, it is crucial to measure upper limb function with standardized outcome measures (OMs). The use of OMs in physical therapy can lead to more efficient rehabilitation programs for the patients and more significant insights into the clinical condition [5,6]. Therefore, the selection of OMs with good psychometric properties is highly recommended. One of the most commonly OMs used by physical therapists and other health care professionals to assess the performance of the upper extremities in people who have suffered a stroke is the Action Research Arm Test (ARAT). The ARAT is a standardized and validated test that consists of 19 movement tasks. The tasks are divided into four main tests, known as subscales (Grasp, Grip, Pinch, and Gross arm movement). The ARAT has demonstrated excellent reliability, and it is a relatively short and simple measure of upper limb function, and testing can be completed quickly on higher functioning patients [7]. ARAT, like most other tests, requires a human examiner to transform observations of a patient’s movement into a score. The reliance on a human examiner leaves room for subjective measures, particularly in scoring, especially patterns of motor test abnormality that emerge after a stroke [8]. In recent years, due to technological development, several investigations have been developed in medical rehabilitation, such as using multiple types of sensors to analyze human hand motion. The most common sensors used for hand motion analysis are data glove, inertial measurement unit (IMU), optical markers, vision-based capturing (Ordinary Cameras, Depth Cameras, Leap Motion Controller), electromyography sensor (EMG)-based capturing, and force sensors (Capacitive, Piezoresistive, Piezoelectric) [9]. The hand motion data obtained from these sensors allow us to know: hand position, finger joint angles, force detection, and angular velocity in real-time. Assessment and analysis of upper extremities in post-stroke patients using diverse types of sensors can be found in several investigations. Lin et al. [10] proposed a data glove system integrated with six-axis inertial measurement unit sensors for evaluating the hand function. A shoe-based sensor with force-sensitive resistors (FSRs) to accurately identify postures in people with stroke was proposed by Fulk and Sazonov [11]. Ambar et al. [12] designed an arm rehabilitation monitoring device utilizing an Arduino-based Microcontroller using a flex sensor to detect arm bending movement, an IMU board (InvenSens Inc., San José, CA, USA) and two force-sensitive resistors to detect muscle force. Data from a Microsoft Kinect sensor (kinematic upper limb) and an FSRs glove (strength of muscles) to predict muscle forces in stroke patients through the least square regression matrix were used by Hoda et al. [13]. A data-glove-based system embedded with 9-axis IMUs sensors and FSRs for evaluation of hand function was designed by Hsiao et al. [14]. Kim et al. [15] proposed a Microsoft Kinect sensor tool during the Fugl–Meyer assessment (FMA) and validated it for hemiplegic stroke patients. Schwarz et al. [16] used a wearable inertial sensing system composed of eight IMUs, with triaxial accelerometers and gyroscopes, to assess upper extremity movement impairments after stroke.

However, to the best of our knowledge, few studies integrate sensors during the execution of the ARAT. Carpinella et al. [17] presented an analysis for quantitative assessment of upper limb motor function in healthy subjects and persons with Multiple Sclerosis, using a single inertial sensor on the wrist. Nam et al. [18] quantified the Range of Motion (ROM) of the upper extremities during the performance of the ARAT and six essential ADL, using 25 inertial measurement unit (IMU) sensors. Ticó Falguera [19] published a study to assess the ROM of finger joints and hand simulation using the CyberGlove II (CyberGlove Systems. LLC, San José, CA, USA) in post-stroke patients during the first six months of recovery. Held et al. [20] designed a study to evaluate rehabilitation progress with a full-body IMU system composed of 14 IMUs, over four weeks. Repnik et al. [21] presented a study on healthy subjects and patients after stroke to quantify upper limb movement, using a wearable system of 7 IMUs for kinematics and electromyography (EMG) sensors for muscle activity analysis. Nevertheless, only one research incorporates multiple sensors and focuses on hand finger joints, but not on finger forces. The use of multisensory information of human hand motion with wearable sensors during a standardized (OMs) performance as the ARAT test is an alternative for a more objective, accurate, and quantitative measurement method. The purpose of this study was to improve the ARAT assessment using a Hand motion system based on a data glove (CyberGlove II^®^) and five force-sensing resistors (FSR). Therefore, in this study, the flexion angles of 11 finger joints and fingertip forces were measured during the performance of three subscales (grasp, grip, and pinch) of the ARAT in healthy subjects to assess hand function. The Gross movement subscale was excluded in this study because it evaluates the motion of the major upper extremity joints (shoulder, elbow, forearm), and the objective of this study was to evaluate the finger joints. In the next stage of the project, the data obtained will be used for clinical purposes as a dataset for machine learning classification algorithms in post-stroke patients.

## 2. Materials and Methods

### 2.1. Measurement System

**Figure 1:**
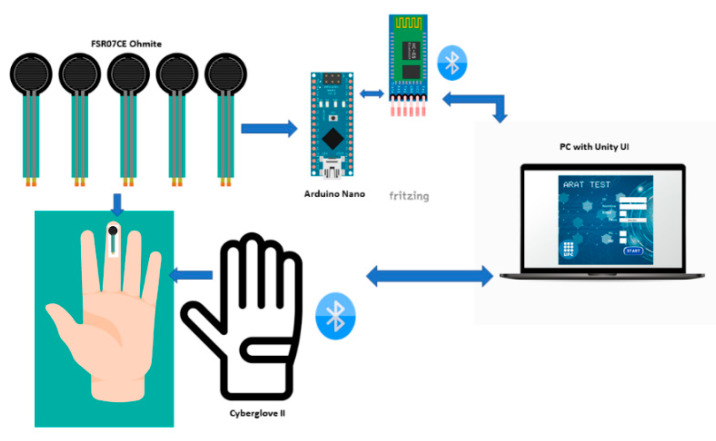
The hand motion system diagram for measuring flexion angles and finger forces.

The design of the Hand motion system is described in this section. The system is composed of a data glove CyberGlove II^®^, Force Sensing Module, and a Graphical User Interface (GUI) developed in Unity^®^ software; the hand motion system diagram is shown in Figure 1. The force sensing module allows measuring individual finger forces and contact points when grasping objects. At the same time, the data glove measures the range of motion of the finger joints. Thus, integrating a force sensing module and a data glove provides a novel hand motion system to assess hand function during the performance of the ARAT activities. The data glove used in this work is the CyberGlove II^®^ which provides up to 22 high-accuracy joint-angle measurements in real-time. The glove has 18 resistive flex sensors and 8-bit digital signal output; the sensor has a resolution: <1 degree and sensor repeatability: 3 degrees [22]. A previously calibrated protocol to convert raw data obtained from the CyberGlove II^®^ in finger joints angles based on a 25 degrees of freedom (DOF) model [23] was used in this study. The procedure to pass the readings of the 18 sensors of the glove to the 25 DOF model is based on linear interpolation, and it is the same for each joint. Since there are DOF that depend on the readings of one or more sensors, the maximum and minimum reading of each sensor and the range of motion (ROM) of each hand joint are considered to obtain each calibration equation [24]. The data glove provided the angles of flexion/extension (F/E) of the distal interphalangeal joint (DIP), proximal interphalangeal joint (PIP), and metacarpophalangeal joints (MCP) of the fingers (index, middle, ring, and pinky) and interphalangeal joint (IP), carpometacarpal joint (CMC) and MCP for the thumb. Data from the glove were transmitted wirelessly to a PC via Bluetooth.

The force sensing module is integrated for five force sensing resistors (FSR); FSRs are devices that allow measuring static and/or dynamic forces applied to a contact surface. We used a commercial model (FSR07CE Ohmite) with an active area diameter of 14.7 mm and 0.375 mm thickness, sensing range 20 g to 5 kg [25]. The FSR sensors were calibrated under static conditions before application to reduce inaccuracies; calibrated weights were statically placed over the sensor to obtain a graph with the relation between the force applied and the output voltage, thus obtaining the equation of the calibration curve. Similar procedures to calibrate FSR have been used in several studies [26,27,28]. Terminals on the FSR were soldered to wires connected to an Arduino Board to convert raw data into force. The Arduino Nano board was used due to its small size, is based on an ATmega328 microcontroller with 8 Analog Input Pins, the PCB Size is 18 × 45 mm, and it weighs only 7 g. The Arduino boards contain a multichannel, 10-bit analog to digital converter (ADC). An Arduino sketch was programmed in the Arduino IDE (Integrated Development Environment), a free software used as a programming platform. The sketch reads the analog inputs voltages, corresponding to the amount of pressure on each FSR sensor and converted into digital voltage. Then, the digital voltage is transformed into a force using the linear equation derived during the calibration process. The force data from Arduino were transmitted via wireless to the GUI; for this purpose, we used the HC-05 module Bluetooth SPP (Serial Port Protocol) because of its compatibility and size. The module has six pins and can easily be interfaced with the Arduino Nano board; the logic voltage level of the data pin is 3.3 V to 5 V [29]. The force sensing module (sensors and signal conditioning hardware) is powered by 9 volts Li-Ion battery. The Arduino Nano and HC-05 are powered by another one, which is sufficient for a minimum of seven hours of data collection.

**Figure 2:**
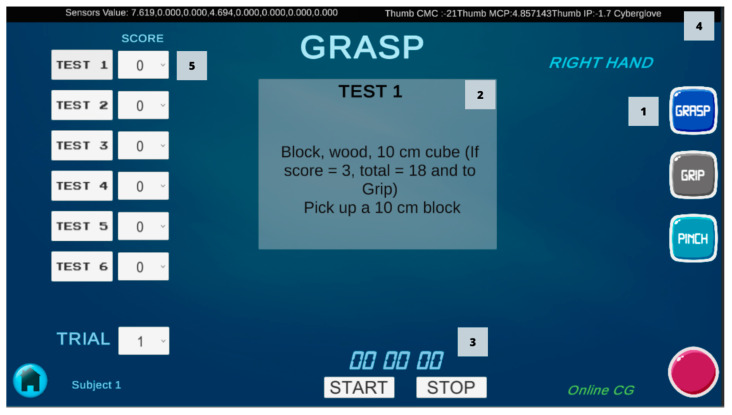
ARAT scene of the Graphical User Interface; (1) Buttons to select each subscale; (2) Description of the test; (3) Buttons to record each test with timer; (4) Sensors information in real-time (angle finger joints and forces); (5) Dropdown list to score each test.

A user-friendly Graphical User Interface (GUI) was developed using the software Unity^®^ version 2020.2.2f1., Unity^®^ is a game-development environment used to create games and applications [30]. Two scenes were designed for the GUI; the first scene contains the main menu with the basic information of patients (name, gender, age, hand length (HL), and hand breadth (HB)). The second scene is the ARAT scene, for the assessment of each patient. In this scene, the information from the sensors is displayed and recorded during the performance of each of the ARAT tests (Figure 2). A script was written in C# using JetBrains Rider 2019.2.3 and attached to the ARAT scene for reading raw data from the CyberGlove and converted in the angles of the finger joints with the calibration equations. Another script was created using an Arduino Bluetooth Plugin to connect the Arduino board and obtain the force data. Data obtained from the sensors in each activity were recorded into a Comma-Separated Values (CSV) file. Data were captured at a rate of 50 Hz and were filtered with a 5-Hz lowpass second-order Butterworth filter in MATLAB^®^ software for statistical analysis.

### 2.2. ARAT Test

The action research arm test ARAT evaluates 19 tests of arm motor function, both distally and proximally. The tests are distributed across four subscales (Grasp, Grip, Pinch, Gross movement), with four to six tasks each. The first three subscales assess the patient’s ability to perform functional tasks, including lifting and moving objects of various shapes and sizes (e.g., blocks, balls, and marbles) [8,31]. The last subscale is the gross arm movement which assesses the movement of the entire upper limb. In each subscale, task performance is scored on a 4-point scale and ordered hierarchically by difficulty to improve testing efficiency. The required materials are a chair without armrests, a table, a 37 cm high shelf, and specific materials. The description of each test and material specifications are shown in Table 1.

### 2.3. Participants

This study included 25 healthy subjects, 14 women and 11 men, whose descriptive data are shown in Table 2. The subjects were selected under the criteria of being right-handed, over 18 years old, and not having suffered any hand disorders or injury. The study was approved by the Ethics Committee of the Polytechnic University of Uruapan (UPU) Michoacán, Mexico. All participants provided written consent after being informed of the aims and procedures of the experiments.

### 2.4. Experimental Setup

**Figure 3:**
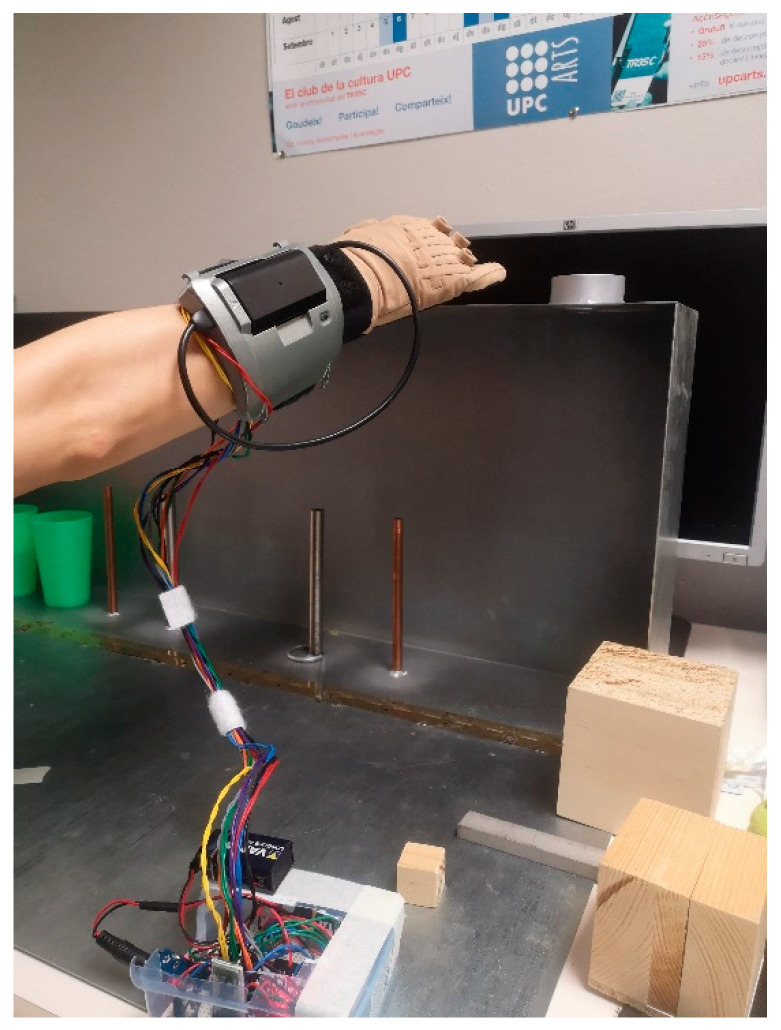
A participant is wearing the hand motion system, performing the Action Research Arm test (ARAT).

The study was performed in the facilities of the UPU. The ARAT equipment for the test performance was prepared according to the instructions and measures obtained from the model described by Lyle [31]. All subjects received verbal and written descriptions for all procedures before the test. Hand length and Hand breadth were measured in each subject with a tape measure. The subjects were equipped with the multisensory system in the right hand; five FSRs were attached to the fingertip of the fingers (thumb, index, middle, ring, little) with a double-sided tape, the force sensing module was placed at one side of the table, a silk glove was placed taking care that the sensor wires pass through the dorsal part of the hand. Finally, the CyberGlove II^®^ was put on in the hand, and the connection Bluetooth with the GUI was tested. Next, the subjects performed the 16 tests part of the subscales: Grasp, Grip, and Pinch, the description of each test are shown in Table 1. Each subject performed the 16 tests three times each (Figure 3). The data were measured and recorded in a CSV file from the start to the end of each activity.

During the beginning of each test, the hand is in a neutral position placed horizontally on the table, so we assumed that this would be the maximum extension angle of the finger joints in many tasks and was not considered in the analysis. Next, at the time of pre-grasp the object, the angle of flexion increases, but no force is exerted. During the grasping event, the maximum flexion angle and maximum force are reached. Finally, when the subject releases the object, the force starts to decrease and the hand back to the start position (extension). Therefore, the maximum values of the three measurements obtained for each subject during each test were averaged. These values were defined as the flexion angle and fingertip force of a subject for a given test. Next, the respective flexion angles and force among the 25 participants were averaged. The finger joints evaluated in this study were as follows: MCP and PIP joints of the fingers (index, middle, ring, and pinky) and CMC, MCP, and IP for the thumb. The distal interphalangeal (DIP) joint was not considered in the analysis because a linear relationship with the proximal PIP joint was assumed, it was considered as DIP=2/3∗PIP adopted from [32]. However, the information of the DIP joint is in the database. The gross movement subscale is excluded in the study because it assesses the entire arm, and the objective of this study is to assess finger joints.

### 2.5. Statistical Analysis

Statistical analysis was conducted using the Software R 4.1.0 and IBM SPSS Statistics Version 28.0. Armonk, NY: IBM Corp. First, the flexion angles of the finger joints were compared among the three subscales (Grasp, Grip, and Pinch), an ANOVA Welch’s test for unequal variances was used to determine whether the differences between group means were statistically significant. In the Pinch subscale, an independent-samples *t*-test was conducted to compare the flexion angle of the finger joints between similar tests using specific fingers but with different objects (ball-bearing, marble). Finally, the mean flexion angles during the 16 tests were compared among the finger joints using a Welch’s ANOVA. Subjects’ age and hand length (HL) were tested for significant differences with respect to the flexion angles using a Mann–Whitney U test. Differences in fingertip force between age groups were analyzed using a Mann–Whitney U test. A Games–Howell post hoc test was conducted for significant differences with a Bonferroni p-adjust correction. The level of significance was set at α = 0.05 for statistical tests.

## 3. Results

### 3.1. Joint Flexion Angles

The flexion angles of the finger joints required to perform each test are shown in Table 3. In the grasp subscale, maximum flexion angles were found during the performance of Test 2 (index MCP 49.9°, middle MCP 50.1°, and ring MCP 39.2°), and Test 6 (thumb CMC 31°, index MCP 52.6°, middle MCP 49.2°, ring MCP 42.2°, index PIP 40.6°, middle PIP 49.5°, and ring PIP 45.2°). In the grip subscale, maximum flexion angles were found in Test 10 (index MCP 58.6°, index PIP 54.7°, middle MCP 57.4°, middle PIP 58.6°, ring MCP 63.5°, ring IP 54.3°, little MCP 52.9°, and PIP 33.6°), Test 8 and Test 9 presented similar flexion angles. In the pinch subscale, flexion angles were similar in the six tests, considering that specific fingers were used in each test.

### 3.2. Pinch Subscale t-Test

An independent-samples *t*-test showed that flexion angle of the Index PIP in Test 12 (M = 43.8, SD = 10.1) was significantly smaller than Test 14 (M = 52, SD = 13.15) conditions; t (48) = −2.46, *p* = 0.017 see Table 4. The flexion angle of the Middle PIP in Test 13 (M = 44.8, SD = 11) was significantly larger than Test 16 (M = 37.3, SD = 8.72) conditions; t (48) = 2.64, *p* = 0.011 see Table 5. Lastly, flexion angle of the Ring PIP in Test 11 (M = 43.5, SD = 11.1) was significantly larger than Test 15 (M = 34.9, SD = 9.82) conditions; t (48) = 2.87, *p* = 0.006 see Table 6.

### 3.3. Grasp, Grip, and Pinch Subscales

**Figure 4:**
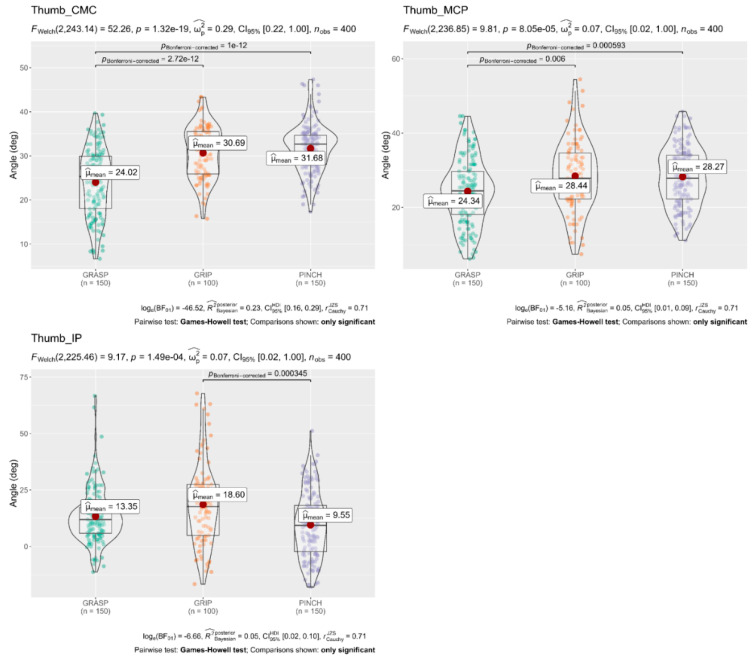
Welch’s ANOVA and post hoc results; Comparison of flexion angles of the Thumb joints concerning the three Subscales of the ARAT. The flexion angles of the carpometacarpal (CMC) and metacarpophalangeal (MCP) joints of the Thumb finger were significantly larger (*p* < 0.05 each) in the Pinch and Grip subscale than in the Grasp subscale. Likewise, the flexion angles of the interphalangeal (IP) were significantly larger (*p* < 0.05 each) in the grip subscale than in the grasp subscale. Horizontal lines indicate significant differences (*p* < 0.05, Games–Howell test post hoc comparison); deg = degrees.

**Figure 5:**
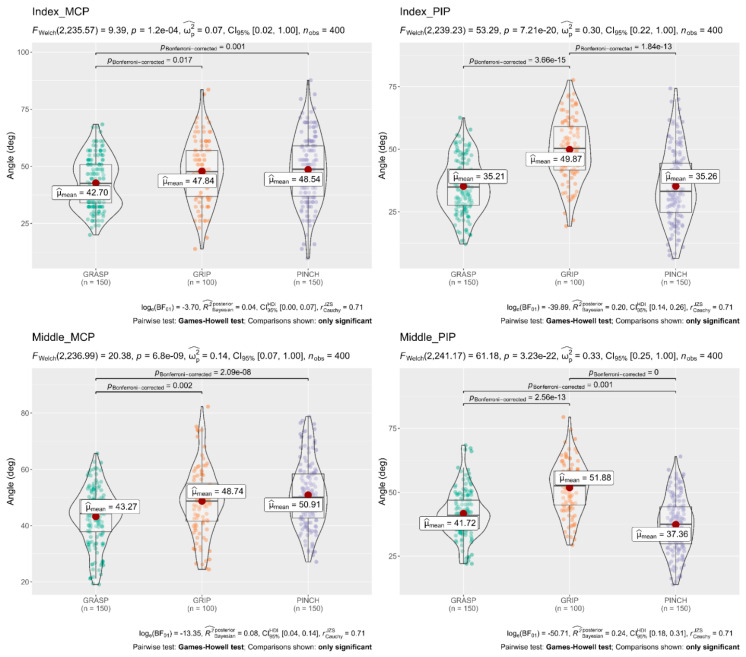
Welch’s ANOVA and post hoc results; Comparison of flexion angles of the Index and Middle joints concerning the three subscales of the ARAT. The flexion angles of the proximal interphalangeal (PIP) joints of the Index and Middle fingers were significantly larger (*p* < 0.05 each) in the grip subscale than in the grasp and pinch subscale. In contrast, the flexion angles of the metacarpophalangeal (MCP) joints were significantly larger (*p* < 0.05 each) in the pinch subscale than in the grasp subscale. Horizontal lines indicate significant differences (*p* < 0.05, Games–Howell test post hoc comparison); deg = degrees.

**Figure 6:**
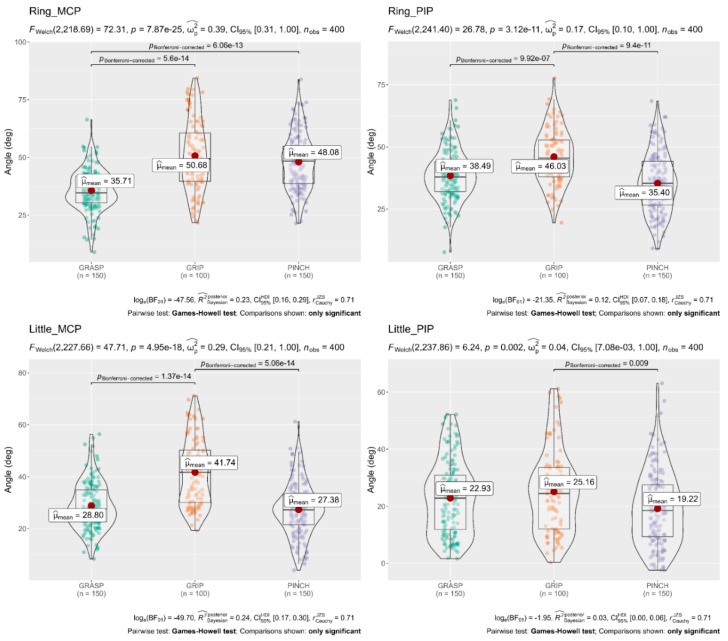
Welch’s ANOVA and Post hoc results; Comparison of flexion angles of the Ring and Little joints concerning the three Subscales of the ARAT. The flexion angles of the metacarpophalangeal (MCP) and proximal interphalangeal (PIP) joints of the Ring finger were significantly larger (*p* < 0.05 each) in the grip subscale than in the grasp subscale. On the other hand, the flexion angles of the metacarpophalangeal (MCP) and proximal interphalangeal (PIP) joints of the Little finger were significantly larger (*p* < 0.05 each) in the grip subscale than in the pinch subscale. Horizontal lines indicate significant differences (*p* < 0.05, Games–Howell test post hoc comparison); deg = degrees.

A Welch’s ANOVA was conducted to determine whether the flexion angle of the finger joints differed based on the different subscales (Grasp, Grip, and Pinch). The results showed that mean flexion angles of the Thumb (CMC 31.68°, MCP 28.27°), Index MCP 48.54°, Middle MCP 50.91°, and Ring MCP 48.08° obtained in the Pinch subscale were significantly larger than flexion angles of the Thumb (CMC 24.02°, MCP 24.34°), Index MCP 42.70°, Middle MCP 43.27°, and Ring MCP 35.71° obtained in the Grasp subscale. In contrast, flexion angles in the Index PIP 49.87°, Middle PIP 51.88°, and Ring PIP 46.03° obtained in the Grip subscale were significantly larger than flexion angles obtained in the Grasp and Pinch subscale. The full results are shown in Figure 4, Figure 5 and Figure 6.

### 3.4. Flexion Angle of Each Finger Joint during the 16 Tests

**Figure 7:**
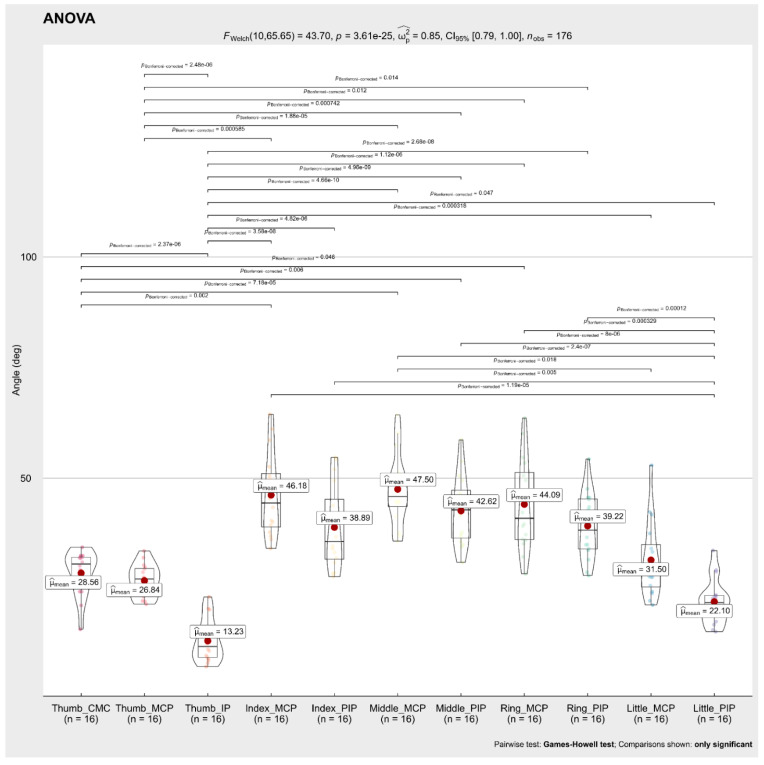
Welch’s ANOVA and Post hoc results; Comparison flexion angles concerning the finger joints, during the 16 tests of the ARAT; Horizontal lines indicate significant differences (*p* < 0.05, Games–Howell test post hoc comparison). The flexion angles of the carpometacarpal (CMC), metacarpophalangeal (MCP), and interphalangeal (IP) joints of the Thumb finger were significantly smaller (*p* > 0.05 each) than the flexion angles of the MCP joints of the Index, Middle, Ring. Similarly, the flexion angles of the proximal interphalangeal (PIP) joint of the Little finger were significantly smaller (*p* > 0.05 each) than the flexion angles of the MCP and PIP joints of the Index, Middle, and Ring fingers. deg = degrees.

A Welch’s ANOVA was conducted to determine whether the mean flexion angle during the 16 ARAT tests differed based on the different finger joints. The results showed no significant differences between the mean flexion angle of the Index MCP 46.18°, Index PIP 38.89°, Middle MCP 47.5°, Middle PIP 42.62°, Ring MCP 44.09°, Ring PIP 39.22° finger joints, while mean flexion angles of the Thumb (CMC 28.56°, MCP 26.84°, IP 13.23°) and Little PIP 22.10° were significantly smaller than the other finger joints. The full results are shown in Figure 7.

### 3.5. Differences in the Flexion Angles Respect to Age and Hand Length Groups

**Figure 8:**
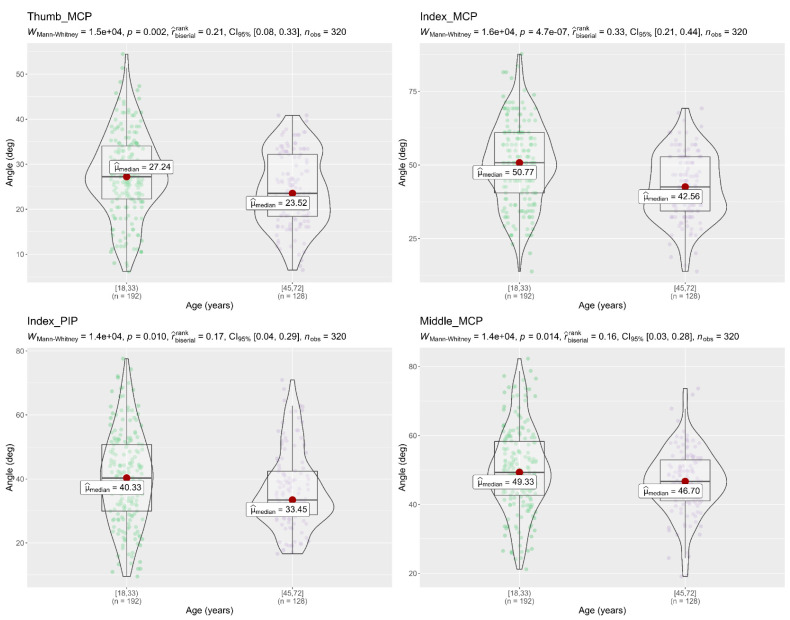
Mann–Whitney U test flexion angle of the finger joints with respect to different age groups. The figure only shows the finger joints in which significant differences were found (Thumb MCP, Index MCP, Index PIP, Middle MCP); *p* < 0.05, significantly different; deg= degrees.

**Figure 9:**
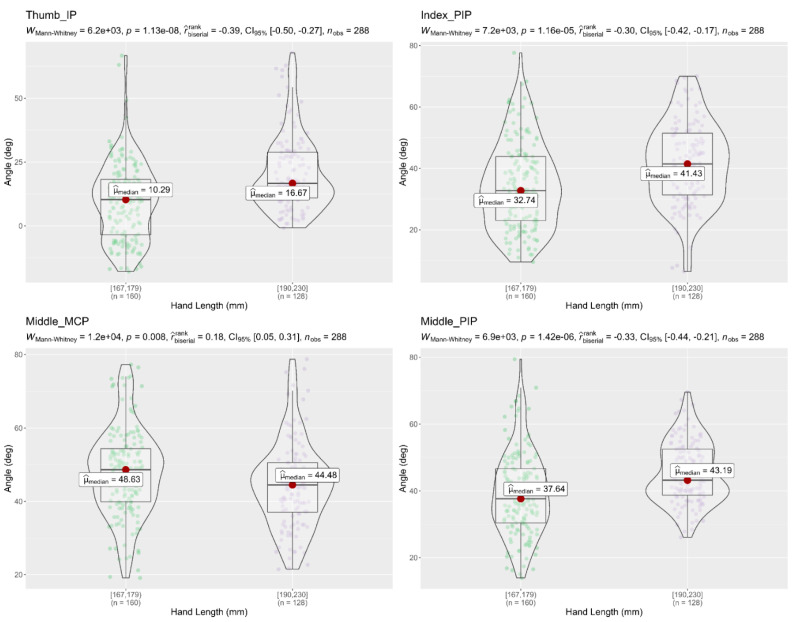
Mann–Whitney U test of flexion angle in finger joints with respect to different hand length groups; The figure only shows the finger joints in which significant differences were found (Thumb IP, Index PIP, Middle MCP, Middle PIP); *p* < 0.05, significantly different; deg = degrees.

**Figure 10:**
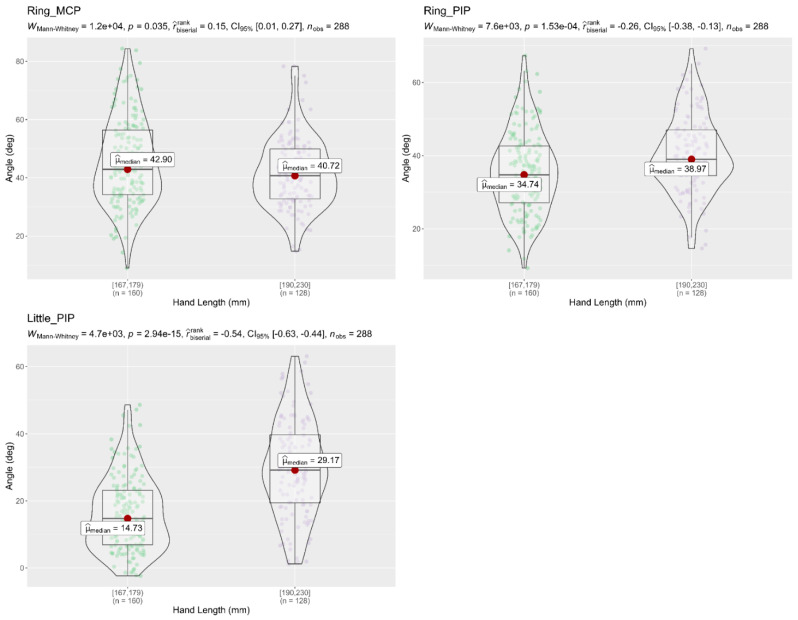
Mann–Whitney U test of flexion angle in finger joints with respect to different hand length groups; The figure only shows the finger joints in which significant differences were found (Ring MCP, Ring PIP, Little PIP); *p* < 0.05, significantly different; deg = degrees.

A Mann–Whitney U test was used to compare the flexion angles of the finger joints between different age groups, and between hand length groups, during the performance of the 16 activities. Results showed that subjects (18–32 years) had a significantly higher flexion angle in the finger joints (Thumb MCP, Index MCP, Index PIP, and Middle MCP) than subjects (45–72 years), as shown in Figure 8. On the other hand, the Mann–Whitney tests in Figure 9 and Figure 10 showed that flexion angles in the Thumb IP, Index PIP, Middle PIP, and Ring PIP in subjects with a hand length of 190–230 mm were larger and statically significant than in subjects with a hand length of 167–178 mm. In contrast, the flexion angles in Middle MCP and Ring MCP were significantly larger in subjects with a hand length of 167–178 mm than subjects with a hand length of 190–230 mm.

### 3.6. Fingertip Forces

The force exerted for the fingertips (thumb, index, middle, ring, and little) during the performance of the 16 tests of the grasp, grip, and pinch subtest are shown in Table 7. During Test 1 of the grasp subscale, the thumb exerts a mean force of 4.5 N, the index exerts a mean force of 2.9 N, the middle exerts a mean force of 3.5 N, the ring exerts a mean force of 2.1 N, and the little exerts a mean force of 1.1 N, with a total force of 14.1 N, these values were the highest of all the tests. In grip subscale maximum total force of 8.1 N was applied in Test 7. Additionally, in the pinch subscale, maximum total forces of 4.5 N and 4.4 N were applied in Test 16 and Test 12, respectively.

The mean force of the fingertips thumb, index, middle, ring, and little fingers required to perform the tests in the Grasp subscale were thumb 2.8 N (1.52), index 2.08 N (1.13), middle 2.16 N (1.2), ring 0.74 N (0.9), and little 0.24 N (0.73). Mean force required to perform grip subscale were thumb 2.39 N (1.5), index 1.95 N (0.94), middle 1.89 N (0.96), and ring 0.39 N (0.63). Finally, the mean forces required to perform the pinch subscale were thumb 2.06 N (0.68), index 2.13 N (0.81), middle 2.02 N (0.69), and ring 1.35 N (0.84).

### 3.7. Differences in Fingertip Force with Respect to Age Groups

An independent-samples *t*-test was conducted to compare the fingertip force exerted for different age groups of subjects during the performance of the tests in grasp subscale. Table 8 shows there was a significant difference in the index finger force of subjects (18–32 years) (M = 1.35, SD = 1.4) and subjects (45–72 years). (M = 0.76, SD = 1.24) conditions; t (118) = 2.38, *p* = 0.019. There was a significant difference in the middle finger force of subjects (18–32 years) (M = 1.77, SD = 1.14) and subjects (45–72 years). (M = 1.14, SD = 1.34) conditions; t (118) = 2.47, *p* = 0.015 see Table 8. Although in the grip subscale there was a significant difference in middle finger force of subjects (18–32 years) (M = 1.28, SD = 1.17) and subjects (45–72 years); (M = 1.14, SD = 1.34) conditions; t (78) = 2.43, *p* = 0.017 see Table 9.

## 4. Discussion

There are only a few studies about the integration of sensors in upper limb measurement methods, but there is a need for more quantitative tests [5,6]. In this paper, we used a hand motion system to determine the flexion angles of the finger joints and fingertip force required to perform the 16 tests of the ARAT in healthy subjects. Traditionally the ARAT is scored on an ordinal four point-scale, that is, from 0 to 3 [31]. A score of 3 is given when the task is performed normally, a score of 2 is given when the subject completes the test but takes a long time or has a difficulty, a score of 1 is given when the subject performs the test partially, a score of 0 is given when the subject cannot perform any part of the test [8,31]. Nevertheless, sometimes the ARAT assessment can be complex and subjective, based on the examiner’s observation and criteria alone. The data obtained indicates that the integration of multiple sensors during the performance of the ARAT allows therapists and other health professionals to perform a more objective, sensitive, and accurate evaluation with a validated clinical test.

To analyze and evaluate the data obtained by the hand motion system, we studied the behavior of each finger joint, from particular to general, during the performance of the 16 tests of the ARAT. The flexion angles were determined for each test presented in Table 3. The flexion angles of the finger joints were compared in each subscale to carry out a more in-depth analysis. In the grasp subscale, flexion angles of finger joints were larger when grasping a small object (Test 2 and Test 6) in comparison to when grasping a larger object (Test 1 and Test 4). In the grip subscale, significant differences were found in Test 7 with respect to the other tests of the subscale. These differences were because the object’s size is larger in this test than in the others.

In the pinch subscale, the results showed that in similar tests using specific fingers but different objects, no significant differences were found in the flexion angle of the thumb joints (CMC, MCP, IP) despite grasping objects of various sizes (Table 4, Table 5 and Table 6). The angle adjustment to make the pinch grip was made by the PIP joint of the other finger involved (index, middle, ring). Similar results where thumb joints did not show significant changes in the flexion angle but index finger joints changed significantly with respect to the object’s width were presented by [33,34].

Next, the subscales of the ARAT were grouped for statistical analysis. Although the subscales are similar, each of them involves different types of grasp, and, therefore, different flexion angles of the finger joints are required. In our study, we observed that flexion angles of the joints MCP in the fingers (index, middle, ring) were larger in tests involving the grasping of small objects, and the pinch subscale involved many of them. Similar results were presented for Lee et al. [35], who found that MCP and PIP joints increased as cylinder diameter decreased, but flexion angles were fairly constant in the DIP joint. Shimawaki et al. [36] found the same correlation using a three-dimensional bone model during the grasping of a cylinder of three different diameters (10, 60, and 120 mm).

Finally, the mean flexion angles obtained during the performing of the 16 activities were as follows: Thumb CMC: 28.56°; Thumb MCP: 26.84°; Thumb IP: 13.23°; Index MCP: 46.18°; Index PIP: 38.89°; Middle MCP: 47.5°; Middle PIP: 42.62°; Ring MCP: 44.09°; Ring PIP: 39.22°; Little MCP: 31.50°; Little PIP: 22.10°. The mean flexion angles of the thumb joints (CMC, MCP, and IP) were significantly smaller than the flexion angles of the index, middle, ring, and little finger (MCP, IP) joints. A similar result was obtained during the performance of 16 activities of daily living (ADL) in the MCP joints of the hand by Murai et al. [37]. On the other hand, no significant differences were found in the mean flexion angle of the index, middle, and ring (MC, PIP) joints, but statistically significant differences were found in the flexion angle of the Little (MCP, PIP) with respect to the other finger joints. Excluding the pinch test in our research, no statistically significant differences between all the MCP fingers joints were found. Hume et al. [38] reported similar results, that there were no significant differences between the flexion angles of the finger joints during 11 ADL [38], while Bain et al. [39] found during the performance of the Sollerman hand grip function test, statistically significant differences between the mean values for the active ROM of the finger joints. The differences found in these studies with our study is that grip and pinch subscales of ARAT involved the radial side of the hand, radial activities include the precision grip between (thumb, index, and middle fingers) and precision pinch between the thumb and the (index or middle or ring) finger. As far as we know, there is little research studying the range of motion of the thumb joints. Hume et al. measured a mean angle of 21 degrees of flexion in the Thumb MCP and 18 degrees in the IP joint during grip postures, and Murai et al. [37] measured 35.3 degrees of flexion in his research. Our study obtained similar results than Hume et al. [38], the flexion angles of the thumb were MCP 26.84° and IP 13.3°. The flexion angles were smaller than Murai et al. because many of the tests in ARAT involves precision grip and pinch.

Regarding to the relationship of the flexion angle of finger joints with respect to different age groups, the results showed that young subjects (18–32 years) had a greater flexion angle in the finger joints (Thumb MCP, Index MCP, Index PIP, and Middle MCP) than elderly subjects (45–72 years) during the performance of the 16 activities. Similar results were presented for Smahel and Klímová [40]. They found that university students had a statistically significantly wider range of motion in the finger joints MCP, PIP, and DIP than Seniors citizens, while DeSmet et al. [41] found a significant correlation between increasing the age and decreasing MCP and IP flexion of the thumb in a study with 101 subjects. Regarding the flexion angles of the finger joints compared to different hand sizes, the result showed that subjects with a longer hand length performed greater flexion in Thumb IP, Index PIP, Middle PIP, and Ring PIP, but had a smaller flexion in Middle MCP and Ring MCP. Similar results were found during an experiment with cylinders of different radius, subjects with the largest HL, close the hand around the object with a slightly larger joint angle by Peña-Pitarch et al. [42].

The fingertip forces for each test are presented in Table 7. Maximum total finger forces were exerted during the performance of Test 1 (14.1 N), Test 4 (7.7 N), and Test 7 (8.1 N). In these tests, a power grip was executed between the fingers and the palm, power grip uses high force but low precision movements and involves the radial and ulnar sides of the hand [43]. Moreover, the results showed that the youngest subjects applied greater strength than the oldest subjects in the index and middle fingertip when performing the six tests part of the grasp subscale and in the middle fingertip during the four tests of the grip subscale. The mean total force in the six tests of the grasp subscale was 8.2 N, grip subscale 6.61 N, and pinch subscale 3.89 N. On the other hand, the force sensor data allows us to know the contact points used to grasp an object. Peña-Pitarch et al. (Peña-Pitarch et al., 2020) found that the number of fingers used to grasp a cylindrical object depends on the radius (ρ) of the object, e.g., for 5 ≤ ρ ≤ 12.5 mm used two fingers, 12.5 ≤ ρ ≤ 20 mm used three fingers, 20 ≤ ρ ≤ 35 mm used four fingers, and 35 ≤ ρ ≤ 70 mm used five fingers. In our study, we found similar results, e.g., when we used objects with a length (*l*) ≤ 100 mm subjects used five fingers, in objects 50 ≤ *l* ≤ 75 mm subjects used four fingers and in objects 10 ≤ *l* ≤ 25 mm were used three fingers. The differences found in our study were due to the object orientation, while in Peña-Pitarch et al., subjects performed a cylindrical grip since the cylinder was in a horizontal position and in this research, the subjects performed a three jaw-chuck grip due to the vertical position of the object.

The limitation in the study was that using a data glove and sensors attached to the fingertips makes it difficult for some subjects to grasp small objects accurately; despite this, all subjects completed the tests. Another limitation is that the test of gross movement was not performed. Therefore, it would be suitable to use a motion capture system based on inertial measurement units (IMU), as were found in other studies [18,21] for measuring flexion angles of the entire arm. In our research, the abduction and adduction angles of the finger joints were not analyzed; these data may be important for evaluating the ARAT and should be considered for future studies.

## 5. Conclusions

The results showed that flexion angles in Thumb (CMC, MCP), Index MCP, Middle MCP, and Ring MCP finger joints obtained in the pinch subscale were significantly larger than flexion angles in the grasp subscale. We determined that the flexion angles depend on the object size and the type of grasp used (power, precision, or pinch). In contrast, the mean total fingertip force exerted on the fingers was significantly greater in the grasp subscale (8.2 N) than in the grip (6.61 N) and the pinch (3.89 N) subscales. The data obtained showed that the integration of multiple sensors during the performance of 16 tests of the ARAT allows therapists and other health professionals to perform a more sensitive, objective, and quantitative assessment of the hand function. Future work will use the data as a dataset for machine learning algorithms with stroke patients.

## Figures and Tables

**Table 1:** Materials and test description of the Action Research Arm Test.

Test	Item (Size)	Description
	**Grasp subscale**	
1	Block, 10 cm^3^	Grasp, lift vertically, place, and release the item onto the top of the shelf.
2	Block, 2.5 cm^3^	
3	Block, 5 cm^3^	
4	Block, 7.5 cm^3^	
5	Cricket ball (Diameter, 7 cm)	
6	Sharpening stone (10.0 × 2.5 × 1 cm)	
	**Grip Subscale**	
7	Two plastic tumblers (Upper diameter, 7 cm; lower diameter, 6 cm; height, 12 cm)	Pour water from one glass into another.
8	Displace alloy tube (Diameter,2.25 cm)	Displace from one side of the table to the other.
9	Displace alloy tube (Diameter,1 cm)	Displace from one side of the table to the other.
10	Put washer over bolt (Diameter, 0.5 cm)	Put washer over the bolt.
	**Pinch subscale**	
11	Ball-bearing (Diameter, 6 mm)	Held the ball-bearing between ring and thumb finger.
12	Marble (Diameter, 1.6 cm)	Held the marble between index and thumb finger.
13	Ball-bearing (Diameter, 6 mm)	Held the ball-bearing between middle and thumb finger.
14	Ball-bearing (Diameter, 6 mm)	Held the ball-bearing between index and thumb finger.
15	Marble (Diameter, 1.6 cm)	Held the marble between ring and thumb finger.
16	Marble (Diameter, 1.6 cm)	Held the marble between middle and thumb finger.

**Table 2:** Descriptive data of the subjects.

Subject Data	Descriptive Statistics			
	Mean	SD	Min	Max
Age (years)	40.2	18.1	18.0	72.0
HL (mm)	176.6	4.4	167.0	184.0
HB (mm)	75.4	3.8	70.0	84.0

HL = Hand length (measured from the tip of the longest finger to the crease under the palm); HB = Hand breadth (measured across the widest area where the fingers join the palm); Standard Deviation (SD); Minimum (Min); Maximum (Max).

**Table 3:** Descriptive statistics of flexion angles required to perform ARAT test.

Test	Thumb			Index		Middle		Ring		Little	
	CMC (deg)	MCP (deg)	IP (deg)	MCP (deg)	PIP (deg)	MCP (deg)	PIP (deg)	MCP (deg)	PIP (deg)	MCP (deg)	PIP (deg)
	Mean (SD)	Mean (SD)	Mean (SD)	Mean (SD)	Mean (SD)	Mean (SD)	Mean (SD)	Mean (SD)	Mean (SD)	Mean (SD)	Mean (SD)
1	15.9	27.6	20.3	36.1	33.2	37	46.5	28.4	45.6	22.3	29.3
	(6.0)	(9.0)	(12.0)	(9.4)	(11.9)	(10.3)	(6.7)	(9.0)	(6.4)	(6.4)	(14.3)
2	27.3	23.8	9.6	49.9	32	50.1	35.7	39.2	31.6	27.6	19.3
	(6.5)	(8.4)	(6.8)	(7.6)	(9.8)	(6.6)	(7.7)	(7.6)	(10.1)	(8.2)	(12.1)
3	24.4	22.2	9.9	40.4	34.6	44.4	37.8	36.2	34.9	27.7	19.6
	(6.4)	(9.5)	(8.0)	(9.4)	(9.6)	(7.7)	(6.5)	(5.3)	(6.8)	(7.5)	(12.4)
4	21.2	23.2	11.3	37.8	36.8	37.6	44.1	32.2	39.9	27.8	22.8
	(6.6)	(9.6)	(10.8)	(9.5)	(12.8)	(9.6)	(7.2)	(8.2)	(8.8)	(6.0)	(13.2)
5	24.3	22.3	13.1	39.4	34.1	41.4	36.7	36	33.7	34.1	23.5
	(5.8)	(8.3)	(10.2)	(10)	(7.9)	(8.9)	(5.5)	(6.0)	(6.1)	(8.7)	(10.5)
6	31.0	27.0	15.8	52.6	40.6	49.2	49.5	42.2	45.2	33.3	23.1
	(4.9)	(7.2)	(17.9)	(9.4)	(9.7)	(7.5)	(10.1)	(9.5)	(10.8)	(11.7)	(12.4)
7	25.7	21.5	15.5	34.1	41.1	35.8	44.5	35.2	36.6	30	20.2
	(5.4)	(8.3)	(12.6)	(10.1)	(9.3)	(7.8)	(6.7)	(9.7)	(7.8)	(5.0)	(11.7)
8	32.1	29.8	15.2	48.1	49.5	50.5	50.5	50.6	45.6	41.8	23.6
	(4.9)	(8.3)	(15.4)	(9.2)	(10.2)	(6.5)	(8.6)	(12.1)	(8.5)	(10.4)	(12.6)
9	32.5	30.4	23.1	50.5	54.2	51.3	53.8	53.4	47.6	42.3	23.2
	(5.5)	(8.9)	(21.4)	(10.3)	(11.1)	(7.1)	(8.8)	(12.3)	(9.1)	(11.2)	(13.4)
10	32.5	32	20.5	58.6	54.7	57.4	58.6	63.5	54.3	52.9	33.6
	(5.5)	(9.2)	(20.6)	(12.4)	(13.8)	(12.6)	(10.5)	(12.1)	(11)	(12.2)	(16.4)
11	34.4	33.5	9.2	36.9	28.4	44.7	41.6	60.1	43.5	37.5	28.9
	(6.0)	(7.4)	(14.9)	(12.7)	(12.1)	(9.7)	(10.9)	(11.5)	(11.2)	(8.5)	(15.7)
12	29.3	24.6	12.6	61.1	43.8	44.8	34.9	39.6	28.7	24.0	17.6
	(5.2)	(6.9)	(16.8)	(8.9)	(10.1)	(7.1)	(10)	(8.6)	(11.2)	(9.9)	(11.7)
13	32	28.7	8.9	45.4	30.9	64.3	44.8	59.5	43	25.7	16.9
	(5.8)	(8.6)	(14.2)	(11.2)	(11.3)	(9.2)	(11)	(9.3)	(11.8)	(9.1)	(11.8)
14	31.9	27.4	11.0	64.4	52	46.9	34.5	37.2	28	21.4	15.7
	(4.4)	(8.3)	(13.5)	(9.6)	(13.2)	(7.0)	(8.9)	(8.1)	(10.5)	(7.3)	(11.5)
15	32.3	29.5	8.2	40.1	27.7	44.6	31	54.7	35.0	31.3	20.9
	(5.4)	(7.2)	(13.5)	(11.5)	(9.8)	(8.2)	(9.1)	(8.8)	(9.8)	(8.7)	(12.5)
16	30.2	25.8	7.4	43.4	28.6	60.1	37.4	47.3	34.1	24.4	15.3
	(5.2)	(7.1)	(14.3)	(10.2)	(9.7)	(8.4)	(8.7)	(8.3)	(9.1)	(8.5)	(10.1)

Test 1–6 corresponds to grasp subscale, Test 7–10 corresponds to grip subscale, Test 11–16 corresponds to Pinch Subscale. CMC = carpometacarpal; MCP = metacarpophalangeal; IP = interphalangeal; PIP = proximal interphalangeal. SD= standard deviation; deg = degrees.

**Table 4:** Independent samples *t*-test for comparison flexion angles with respect to Test 12 and Test 14.

Finger Joints	Levene’s Test		*t*-Test for Equality of Means		
	F	Sig.	t	df	*p*-Value
Thumb CMC	0.78	0.383	−1.95	48	0.58
Thumb MCP	1.14	0.291	−1.27	48	0.21
Thumb IP	0.74	0.394	0.38	48	0.71
Index MCP	0.13	0.719	−1.26	48	0.21
Index PIP	0.96	0.333	−2.46	48	0.017 **

** *p* < 0.05, considered statistically significant.

**Table 5:** Independent samples *t*-test for comparison flexion angles with respect to Test 13 and Test 16.

Finger Joints	Levene’s Test		*t*-Test for Equality of Means		
	F	Sig.	t	df	*p*-Value
Thumb CMC	0.175	0.678	1.135	48	0.26
Thumb MCP	1.315	0.257	1.319	48	0.19
Thumb IP	0.08	0.929	0.353	48	0.73
Middle MCP	0.872	0.355	1.658	48	0.10
Middle PIP	2.062	0.157	2.647	48	0.011 **

** *p* < 0.05, considered statistically significant.

**Table 6:** Independent samples *t*-test for comparison flexion angles with respect to Test 11 and Test 15.

Finger Joints	Levene’s Test		*t*-Test for Equality of Means		
	F	Sig.	t	df	*p*-Value
Thumb CMC	0.27	0.60	1.29	48	0.20
Thumb MCP	0.08	0.78	1.95	48	0.06
Thumb IP	0.26	0.61	0.24	48	0.81
Ring MCP	1.61	0.21	1.87	48	0.07
Ring PIP	0.73	0.40	2.87	48	0.006 **

** *p* < 0.05, considered statistically significant.

**Table 7:** Descriptive statistics of fingertip forces during the performance of the ARAT test.

Test	Thumb Force (N)		Index Force (N)		Middle Force (N)		Ring Force (N)		Little Force (N)		Total Force (N)
	Mean	SD	Mean	SD	Mean	SD	Mean	SD	Mean	SD	
1	4.5	2.1	2.9	1.5	3.5	1.7	2.1	1.1	1.1	1.3	14.1
2	2.3	0.7	2.2	1.1	1.8	0.7	-	-	-	-	6.3
3	2.4	1.1	1.7	0.6	1.5	0.6	0.5	0.7	-	-	6.1
4	2.6	0.9	1.9	1.0	1.8	0.7	1.1	0.8	-	-	7.7
5	2.6	1.2	1.6	0.8	1.9	0.6	0.4	0.6	-	-	6.6
6	2.2	0.9	2.2	1.1	2.5	0.7	-	-	-	-	6.9
7	3.0	1.8	2.0	0.7	2.3	1.1	0.8	0.8	-	-	8.1
8	2.3	1.1	2.1	1.0	1.9	0.8	-	-	-	-	6.4
9	2.1	1.0	1.9	1.1	1.8	0.8	-	-	-	-	6.1
10	2.1	1.0	1.9	0.9	1.7	1.0	-	-	-	-	5.8
11	1.8	0.2	-	-	-	-	1.2	1.0	-	-	3.0
12	2.1	0.7	2.4	0.9	-	-	-	-	-	-	4.4
13	1.8	0.2	-	-	1.8	0.5	-	-	-	-	3.5
14	2.2	0.5	1.9	0.6	-	-	-	-	-	-	4.1
15	2.3	1.0	-	-	-	-	1.5	0.9	-	-	3.8
16	2.2	0.9	-	-	2.3	0.8	-	-	-	-	4.5

Test 1–6 corresponds to grasp subscale; Test 7–10 corresponds to grip subscale; Test 11–16 corresponds to pinch subscale; Force N = Newton.

**Table 8:** Independent samples *t*-test for comparison finger forces in grasp subscale, with respect to different groups of age (18–32 years) and (45–72 years).

Fingertip	Levene’s Test		*t*-Test for Equality of Means		
	F	Sig.	t	df	*p*-Value
Thumb	1.715	0.193	0.807	118	0.421
Index	1.314	0.254	2.385	118	0.019 **
Middle	0.038	0.846	2.477	118	0.015 **
Ring	1.421	0.236	0.662	117	0.510
Little	0.013	0.908	0.051	118	0.959

** *p* < 0.05, significant difference.

**Table 9:** Independent samples *t*-test for comparison finger force in grip subscale, with respect to different groups of age (18–32 years) and (45–72 years).

Fingertip	Levene’s Test		*t*-Test for Equality of Means		
	F	Sig.	t	df	*p*-Value
Thumb	3.152	0.080	1.628	78	0.108
Index	0.247	0.621	0.534	78	0.595
Middle	2.913	0.092	2.429	78	0.017 **
Ring	0.339	0.562	0.618	78	0.538

** *p* < 0.05, significant difference.

## Data Availability

The data presented in this study are openly available in Figshare at https://doi.org/10.6084/m9.figshare.19368008.v1.

## References

[1] Maw J., Wong K.Y., Gillespie P. (2016). Hand Anatomy. Br. J. Hosp. Med..

[2] Dolan D. (2014). Hope Through Research. Neurol. Now.

[3] Feigin V.L., Brainin M., Norrving B., Martins S., Sacco R.L., Hacke W., Fisher M., Pandian J., Lindsay P. (2022). World Stroke Organization (WSO): Global Stroke Fact Sheet 2022. Int. J. Stroke.

[4] Whitehead S., Baalbergen E. (2019). Post-Stroke Rehabilitation. S. Afr. Med. J..

[5] Santisteban L., Térémetz M., Bleton J.P., Baron J.C., Maier M.A., Lindberg P.G. (2016). Upper Limb Outcome Measures Used in Stroke Rehabilitation Studies: A Systematic Literature Review. PLoS ONE.

[6] Sullivan J.E., Crowner B.E., Kluding P.M., Nichols D., Rose D.K., Yoshida R., Pinto Zipp G. (2013). Outcome Measures for Individuals with Stroke: Process and Recommendations from the American Physical Therapy Association Neurology Section Task Force. Phys. Ther..

[7] Teasell R., Hussein N., Mirkowski M., Vanderlaan D., Saikaley M., Longval M., Iruthayarajah J. (2020). Stroke Rehabilitation Clinician Handbook 2020.

[8] Yozbatiran N., Der-Yeghiaian L., Cramer S.C. (2008). A Standardized Approach to Performing the Action Research Arm Test. Neurorehabil. Neural Repair.

[9] Xue Y., Ju Z., Xiang K., Chen J., Liu H. (2019). Multimodal Human Hand Motion Sensing and Analysis-A Review. IEEE Trans. Cogn. Dev. Syst..

[10] Lin B.-S., Hsiao P.-C., Yang S.-Y., Su C.-S., Lee I.-J. (2017). Data Glove System Embedded With Inertial Measurement Units for Hand Function Evaluation in Stroke Patients. IEEE Trans. Neural Syst. Rehabil. Eng..

[11] Fulk G.D., Sazonov E. (2011). Using Sensors to Measure Activity in People with Stroke. Top. Stroke Rehabil..

[12] Ambar R., Ahmad M., Mohd Ali A., Abdul Jamil M. (2011). Arduino Based Arm Rehabilitation Assistive Device. J. Eng. Technol..

[13] Hoda M., Hoda Y., Hafidh B., El Saddik A. (2018). Predicting Muscle Forces Measurements from Kinematics Data Using Kinect in Stroke Rehabilitation. Multimed. Tools Appl..

[14] Hsiao P.C., Yang S.Y., Lin B.S., Lee I.J., Chou W. Data Glove Embedded with 9-Axis IMU and Force Sensing Sensors for Evaluation of Hand Function. Proceedings of the 2015 37th Annual International Conference of the IEEE Engineering in Medicine and Biology Society (EMBC).

[15] Kim W.S., Cho S., Baek D., Bang H., Paik N.J. (2016). Upper Extremity Functional Evaluation by Fugl-Meyer Assessment Scoring Using Depth-Sensing Camera in Hemiplegic Stroke Patients. PLoS ONE.

[16] Schwarz A., Bhagubai M.M.C., Wolterink G., Held J.P.O., Luft A.R., Veltink P.H. (2020). Assessment of Upper Limb Movement Impairments after Stroke Using Wearable Inertial Sensing. Sensors.

[17] Carpinella I., Cattaneo D., Ferrarin M. (2014). Quantitative Assessment of Upper Limb Motor Function in Multiple Sclerosis Using an Instrumented Action Research Arm Test. J. Neuroeng. Rehabil..

[18] Nam H.S., Lee W.H., Seo H.G., Kim Y.J., Bang M.S., Kim S. (2019). Inertial Measurement Unit Based Upper Extremity Motion Characterization for Action Research Arm Test and Activities of Daily Living. Sensors.

[19] Ticó Falguera N. (2016). Biomecànica dels Dits de la mà com a Factor Pronòstic de la Recuperació Funcional de L’extremitat Parètica en Pacients amb Ictus Aguts.

[20] Held J.P.O., Klaassen B., Eenhoorn A., van Beijnum B.J.F., Buurke J.H., Veltink P.H., Luft A.R. (2018). Inertial Sensor Measurements of Upper-Limb Kinematics in Stroke Patients in Clinic and Home Environment. Front. Bioeng. Biotechnol..

[21] Repnik E., Puh U., Goljar N., Munih M., Mihelj M. (2018). Using Inertial Measurement Units and Electromyography to Quantify Movement during Action Research Arm Test Execution. Sensors.

[22] CyberGlove Systems Inc.© CyberGlove II. http://www.cyberglovesystems.com/cyberglove-ii.

[23] Peña-Pitarch E., Falguera N.T., Yang J. (2014). Virtual Human Hand: Model and Kinematics. Comput. Methods Biomech. Biomed. Engin..

[24] Peña-Pitarch E., Costa J.V., Martinez J.L., Al Omar A., Larrión I.A., Tico-Falguera N. (2018). Introductory Analysis of Human Upper Extremity After Stroke. Int. J. Priv. Health Inf. Manag..

[25] Ohmite FSR Series (Datasheet). https://www.ohmite.com/assets/docs/res_fsr.pdf.

[26] Flórez J.A., Velásquez A. Calibration of Force Sensing Resistors (Fsr) for Static and Dynamic Applications. Proceedings of the 2010 IEEE ANDESCON.

[27] Hsu W.C., Sugiarto T., Chen J.W., Lin Y.J. (2018). The Design and Application of Simplified Insole-Based Prototypes with Plantar Pressure Measurement for Fast Screening of Flat-Foot. Sensors.

[28] Ye Q., Seyedi M., Cai Z., Lai D.T.H. (2015). Force-Sensing Glove System for Measurement of Hand Forces during Motorbike Riding. Int. J. Distrib. Sens. Netw..

[29] ITead Studio HC—05—Bluetooth to Serial Port Module (Datasheet). https://datasheetspdf.com/pdf-file/1418730/ITead/HC-05/.

[30] Unity Technologies Unity User Manual 2020.3 (LTS). https://docs.unity3d.com/Manual/index.html.

[31] Lyle R.C. (1981). A Performance Test for Assessment of Upper Limb Function in Physical Rehabilitation Treatment and Research. Int. J. Rehabil. Res..

[32] Rijpkema H., Girard M. Computer Animation of Knowledge-Based Human Grasping. Proceedings of the 18th annual conference on Computer graphics and interactive techniques, SIGGRAPH 1991.

[33] Lee J.W., Rim K. (1991). Measurement of Finger Joint Angles and Maximum Finger Forces during Cylinder Grip Activity. J. Biomed. Eng..

[34] Shimawaki S., Murai T., Nakabayashi M., Sugimoto H. (2019). Measurement of Flexion Angle of the Finger Joint during Cylinder Gripping Using a Three-Dimensional Bone Model Built by X-Ray Computed Tomography. Appl. Bionics Biomech..

[35] Shimawaki S., Nakamura Y., Nakabayashi M., Sugimoto H. (2020). Flexion Angles of Finger Joints in Two-Finger Tip Pinching Using 3D Bone Models Constructed from X-ray Computed Tomography (CT) Images. Appl. Bionics Biomech..

[36] Yokogawa R., Hara K. (2004). Manipulabilities of the Index Finger and Thumb in Three Tip-Pinch Postures. J. Biomech. Eng..

[37] Murai T., Uchiyama S., Nakamura K., Ido Y., Hata Y., Kato H. (2018). Functional Range of Motion in the Metacarpophalangeal Joints of the Hand Measured by Single Axis Electric Goniometers. J. Orthop. Sci..

[38] Hume M.C., Gellman H., McKellop H., Brumfield R.H. (1990). Functional Range of Motion of the Joints of the Hand. J. Hand Surg. Am..

[39] Bain G.I., Polites N., Higgs B.G., Heptinstall R.J., McGrath A.M. (2015). The Functional Range of Motion of the Finger Joints. J. Hand Surg. Eur. Vol..

[40] Smahel Z., Klímová A. (2004). The Influence of Age and Exercise on the Mobility of Hand Joints: 1: Metacarpophalangeal Joints of the Three-Phalangeal Fingers. Acta Chir. Plast..

[41] De Smet L., Urlus M., Spriet A., Fabry G. (1993). Metacarpophalangeal and Interphalangeal Flexion of the Thumb: Influence of Sex and Age, Relation to Ligamentous Injury. Acta Orthop. Belg..

[42] Peña-Pitarch E., Magaña J.F.P., Ticó-Falguera N., Al Omar A., Larrión I.A., Costa J.V. (2020). Virtual Human Hand: Grasps and Fingertip Deformation. Adv. Intell. Syst. Comput..

[43] Cech D.J. (2012). Chapter 14—Prehension. Functional Movement Development Across the Life Span.

